# Editorial: Molecular links between mitochondrial damage and human neurodegenerative disorders, volume II

**DOI:** 10.3389/fcell.2024.1417802

**Published:** 2024-04-29

**Authors:** Yuzuru Imai

**Affiliations:** Department of Research for Parkinson’s Disease, Juntendo University Graduate School of Medicine, Tokyo, Japan

**Keywords:** mitochondria, mtDNA, mitophagy, CHCHD2, CHCHD10, fragile X syndrome, Parkin (PARK2), FMR1

Mitochondria have various functions in cells, including energy production, iron and lipid metabolism, regulation of Ca^2+^ dynamics, and cell death and inflammatory signaling. Dysregulation and degeneration of mitochondria have been shown to cause a variety of neurodegenerative diseases. In 2021, we launched a Research Topic entitled “*Molecular Links between Mitochondrial Damage and Human Neurodegenerative Disorders*,” to which many excellent papers were contributed. This Research Topic consists of two original articles and two review articles that were submitted after 2022 on the same Research Topic. These manuscripts cover mitochondrial metabolism, mitochondrial DNA, membrane potential, and mitochondrial membrane structure.

Fragile X syndrome, one of the triplet repeat diseases, is a disease characterized by developmental delay/intellectual disability beginning in childhood due to mutations in the *Fragile X messenger ribonucleoprotein 1* (*FMR1*) gene. The triplet repeat sequence (CGG) of the *FMR1* gene on the long arm of the X chromosome is extended with each generation. When the number of CGG repeats exceeds 200, Fragile X syndrome develops. Reduced expression of the *FMR1* gene product Fragile X Messenger Ribonucleoprotein (FMRP) is believed to be the core pathophysiology of fragile X syndrome. FMRP, an RNA-binding protein, binds to mRNA and negatively regulates local translation of mRNA in neurons. Using primary cortical neuronal cultures from *FMR1* knockout mice, Bülow et al. report that neuronal activity-dependent changes in mitochondrial morphology and membrane potential differ in dendrites and axon initial segment. The mechanism by which FMRP regulates mitochondria has yet to be determined. *FMR1* knockout mice exhibit elevated protein synthesis (Bhattacharya et al., 2012). Metformin inhibits protein translation by blocking mammalian/mechanistic target of rapamycin complex 1 (mTORC1) and suppresses an autism spectrum disorder-like phenotype in *FMR1* knockout mice (Gantois et al., 2017). Metformin also inhibits mitochondrial respiratory chain complex I and activates AMP-activated protein kinase signaling (Foretz et al., 2014). This mechanism may provide clues for the elucidation of mitochondrial regulation by FMRP.

Mitochondrial DNA deletion syndrome (MDS) is a group of diseases characterized by defects in mitochondrial DNA (mtDNA) maintenance (Basel, 2020). The mitochondrial genome encodes 13 proteins, all of which are important catalytic subunits of the electron transfer chain. Thus, depletion of mitochondrial DNA results in impaired oxidative phosphorylation. Dombi et al. determine the most effective nucleoside supplementation method (optimal concentration and nucleoside combination) based on improving mtDNA content, cell viability, and mitochondrial functions using MDS fibroblasts harboring mutations in *POLG*, *DGUOK*, and *TWNK* (genes encoding alpha subunits of mtDNA γ polymerase, deoxyguanosine kinase, and Twinkle mtDNA helicase, respectively) (Bulst et al., 2009). The findings of this study will be useful for future studies of nucleoside supplementation methods in MDS animal models.

Mitophagy, autophagy of mitochondria, is a physiological phenomenon that is highly conserved from yeast to humans. Mitophagy is observed in metabolic changes during development and differentiation and in the quality control of defective mitochondria. The relationship between neurodegeneration and mitophagy was first discovered in a series of studies that showed that *PRKN* and *PINK1*, the genes responsible for juvenile Parkinson’s disease (PD), are involved in mitophagy (Imai, 2020). Since then, many molecules associated with mitophagy have been identified and PINK1-Parkin (the gene product of *PRKN*)-independent mitophagy pathways have also been reported. Furthermore, mitophagy research continues to expand with reports of its involvement in neurodegenerative diseases such as Alzheimer’s disease and amyotrophic lateral sclerosis (ALS), as well as Parkinson’s disease. Jetto et al. provide an overview of mitophagy signaling in neurodegenerative diseases, including some unresolved issues.

*CHCHD2* and *CHCHD10* have been identified as causative genes for PD and ALS/frontotemporal lobe dementia (FTD), respectively (Bannwarth et al., 2014; Funayama et al., 2015). These two proteins are paralogous to each other, forming a complex in the mitochondrial intermembrane space. CHCHD2 and CHCHD10 have been implicated in maintaining mitochondrial respiratory chain complex function and matrix structure, as well as in the regulation of stress response and cell death. Despite this twin-like relationship, missense mutations in each gene cause different diseases. Ikeda et al. outlined the CHCHD2/CHCHD10 studies reported to date and aimed to provide clues to unravel this mystery.

The multifaceted roles of mitochondria and the involvement of mitochondria in various diseases remain a fascinating Research Topic. Why do specific genes in ubiquitously distributed mitochondria cause specific diseases? To address this question, many researchers are expected to continue work in this field.
